# Path to full immunisation coverage, role of each vaccine and their importance in the immunisation programme: a cross-sectional analytical study of India

**DOI:** 10.1136/bmjph-2024-001290

**Published:** 2025-03-23

**Authors:** Pritu Dhalaria, Pawan Kumar, Sanjay Kapur, Ajeet Kumar Singh, Ajay Kumar Verma, Disha Agarwal, Bhupendra Tripathi, Gunjan Taneja

**Affiliations:** 1 Immunization Technical Support Unit, Ministry of Health & Family Welfare, Delhi, India; 2 Immunization Division, Ministry of Health & Family Welfare, Delhi, India; 3 John Snow India, Delhi, India; 4 Department of Economics, Banaras Hindu University, Varanasi, India; 5 Bill & Melinda Gates Foundation, New Delhi, India

**Keywords:** Public Health, Community Health, Cross-Sectional Studies

## Abstract

**Introduction:**

Immunisation is vital in preventing infectious diseases and promoting public health. This study examines the immunisation landscape in India, focusing on absolute zero dose (defined as a child did not receive any single dose of vaccine as per the National Immunisation Schedule), antigen-wise zero dose (defined as children who did not receive any dose of specific vaccine but received some or complete dose of other vaccines), the pattern of undervaccination (defined as children who missed any one or more than one dose of vaccine from total eight doses of vaccine (one dose-BCG, three doses-DPT, three doses-OPV and one dose-measles vaccine) and immunisation cascade.

**Methods:**

Using data from the National Family Health Survey-5, we analysed the immunisation status of 43 247 children across India. The prevalence of absolute zero-dose children, antigen-wise zero dose, co-coverage rates and cascade levels for vaccine combinations are assessed. The multilevel regression model has been applied to understand the likelihood of left-out and antigen-wise zero doses by socioeconomic determinants.

**Results:**

Children lacking vaccination cards experience a higher prevalence of absolute zero dose cases (21.2%). Notably, scheduled tribes (4.1%), the Muslim group (5.4%) and the poorest wealth quintile (4.6%) exhibit the highest prevalence. Remarkably, within partially vaccinated (20%) children, 42.8% show zero dose for measles-containing vaccines, while 6.7% of children failed to achieve full immunisation coverage due to just one missed dose of vaccine. Further, 20% of the partially vaccinated subset revealed that 7.29% missed full immunisation coverage due to oral polio vaccine (OPV) dose gaps.

**Conclusions:**

Targeted efforts are essential to bridge immunisation gaps and achieve universal coverage in India. Focusing on antigen-specific zero dose and partially vaccinated children, particularly those missing OPV doses and measles vaccine offers the potential to improve full immunisation coverage. Therefore, to achieve the IA2030 requires an intensified target for reaching absolute zero and antigen-wise zero dose.

**WHAT IS ALREADY KNOWN ON THIS TOPIC**

- Recently, there has been a growing emphasis on zero-dose children—those who have not received any vaccines—with a particular focus on achieving the objectives outlined in the Immunisation Agenda 2030. Prior studies, both global and in India, have primarily focused on understanding the trends, patterns and prevalence of zero-dose occurrences, analysing them through various socioeconomic and demographic lenses.

**WHAT THIS STUDY ADDS**

- This study analyses the concept of absolute zero dose, antigen-specific zero doses for antigens and the journey of zero dose to full immunisation coverage through the immunisation cascade.
- Through meticulous mapping of unvaccinated and undervaccinated children in India, this research provides critical insights into areas of concern that require targeted attention.
- A noteworthy aspect of this study lies in its comprehensive exploration of the transformative process that children undergo, transitioning from zero-dose vulnerability to the coveted realm of full immunisation.
- The importance of each vaccine brings attention to immunity gaps against targeted pathogens, which emphasises universal access to vaccines and leaving no child behind.

**HOW THIS STUDY MIGHT AFFECT RESEARCH, PRACTICE OR POLICY**

- The trajectory of this study extends the boundaries of previous research, shedding light on zero-dose scenarios and the transformative journey from zero dose towards full immunisation, necessitating unwavering momentum.
- The nuanced insights from understanding absolute zero dose scenarios and their link to full immunisation serve as a crucial foundation for targeted health system interventions.
- These understandings are pivotal in integrating previously unreached children into the comprehensive fabric of immunisation efforts to reach absolute zero dose and antigen-specific zero-dose children, and with slight effort, they can be converted to fully immunised children and will be crucial in closing the population immunity gap.

## Introduction

 Immunisation stands as a cornerstone of public health, playing a pivotal role in preventing the spread of infectious diseases and safeguarding the well-being of individuals, communities and entire population. Vaccines have helped eradicate or significantly reduce the incidence of numerous devastating diseases throughout history by stimulating the immune system to recognise and fight off specific pathogens.^1 2^ Achieving universal immunisation coverage is a global priority outlined in the Sustainable Development Goals. Aligned with these goals, the Immunisation Agenda 2030 (IA 2030) has set ambitious targets to accelerate progress and ensure that no one is left behind in immunisation efforts.^3^ Despite the remarkable achievements in global immunisation, challenges remain in achieving universal coverage and reaching vulnerable populations.^4^ Socioeconomic disparities, limited healthcare infrastructure, geographical barriers and cultural beliefs significantly hinder immunisation uptake, especially in marginalised and underserved communities.^4^,^7^ Additional social factors, such as limited awareness about vaccination and low education levels among caregivers, contribute to exclusion and low vaccination uptake.^7^,^10^ It is likely that a significant portion of these children belong to specific groups, such as migrants in unmapped areas or those residing in difficult geographical terrains.^8 11^ These factors can limit their access to healthcare services and reduce awareness about the importance of vaccination, as migrant population often face challenges in accessing consistent healthcare, including immunisation services.^12^,^14^

Vaccine uptake in India is shaped by individual beliefs, social dynamics and behavioural patterns, all influenced by diverse cultural factors. Understanding these complexities is essential for designing effective vaccination strategies tailored to India’s diverse sociocultural landscape.^15 16^

The vigilance against vaccine-preventable diseases is emphasised in the context of full immunisation coverage (FIC) before the completion of the first year of life. Missing even a single dose places children at continued risk. The distinction between one-dose vaccines (BCG and measles) and three-dose vaccines (like oral polio vaccine (OPV) and diphtheria-tetanus-pertussis (DPT)) underscores the critical role of complete vaccination and the need to address antigen-specific zero-dose scenarios.^17^

Global data indicate that the prevalence of partially vaccinated children is more than that of unvaccinated children. A recent study conducted by Bianca *et al* covering data from 92 countries revealed that 31.3% of children remain undervaccinated despite having received their first vaccine.^11^ Therefore, it is essential to examine the specific vaccines that children missed, unravel the immunisation cascade’s dynamics and understand the journey of immunisation coverage from zero dose to full immunisation.

Vaccination not only prevent diseases such as diarrhoea, measles, pneumonia, polio and whooping cough but also contribute to broader advancements in education and economic development. According to Ozawa *et al*, a vaccination programme across 72 low-income countries could potentially save 6.4 million lives, prevent 426 million cases of illness and yield cost savings of US$6.2 billion in treatment expenses and US$145 billion in productivity losses from 2011 to 2020.^18 19^

A growing body of research on the impact of measles vaccination on academic achievement and cognitive development has been consistently highlighted across various settings. Studies conducted in South Africa (Anekwe *et al*) and a longitudinal cohort study spanning Ethiopia, India and Vietnam (Nandi *et al*) indicate that timely measles vaccination is associated with higher academic achievement in elementary school, as well as improved cognition and school performance between ages 7 and 12 years.^20 21^ These findings underscore the critical importance of measles zero-dose vaccination.^22^

Children who received hepatitis B and DPT vaccinations during childhood, whether or not the formulations contained thimerosal, scored higher on the Wechsler Intelligence Scale for Children-Revised, as shown in research by Mrozek-Budzyn *et al*.^23^ Additionally, studies indicate that fully vaccinated children demonstrate superior cognitive abilities compared with their partially vaccinated and unvaccinated (absolute zero dose) children.^24^ Global literature suggests that the barriers to immunisation faced by children who have never received a single dose of DPT or OPV may differ from those encountered by children who have initiated but not completed the full vaccination series.^25^

A comprehensive review of global grey literature by Favin *et al* identified multiple factors contributing to incomplete vaccination. These included unfavourable experiences encountered at immunisation centres, such as caregiver mistreatment, prolonged waiting times and drug shortages. Additionally, missed opportunities, such as health workers declining to vaccinate sick children or turning away those without vaccination cards, along with concerns about potential side effects and limited awareness of vaccination schedules, were identified as significant barriers to achieving complete immunisation coverage.^26^

The timing of the vaccine dose, particularly the measles vaccine, typically administered at ages 9–12 months, during the fourth immunisation visit, plays a crucial role in achieving FIC by a child’s first year. Common barriers to vaccinating children include a lack of awareness about vaccine benefits and schedules, distance to vaccination sites and time constraints.^27^

Predictors of non-vaccination and drop-out (absolute zero dose, partially vaccinated, antigen-specific zero dose) between vaccine doses in India have not been systematically studied, highlighting the need for further research to address these critical gaps in immunisation programmes. Effectively reducing undervaccination requires a comprehensive exploration of the diverse risk factors associated with incomplete vaccination (partial doses, antigen-wise zero dose) and non-vaccination (absolute zero dose).

India, having the world’s largest birth cohort, faces unique challenges, as even a small percentage of absolute zero dose or partially vaccinated children can contribute to significant numbers compared with other countries.^28^ The National Family Health Survey (NFHS), a repeated cross-sectional national survey and a key source of information on immunisation coverage in India, shows that India has made significant strides in improving FIC in recent years. The NFHS-5 (2019–2021) has reported FIC as 76.4%, which is a remarkable improvement from FIC reported in NFHS-4 (2015–2016), that is, 62%.^29 30^ Moreover, the healthcare system has successfully reached 96.4% of children, whereas only 3.6% were completely unvaccinated (absolute zero dose). However, it is important to delve deeper into the 20.0% of children who are partially vaccinated (underimmunised, antigen-wise zero dose) and understand the vaccination status of these partially vaccinated children. It is also essential to understand the socioeconomic factors contributing to Absolute zero dose.

Understanding the antigen-wise gaps is crucial for designing targeted strategies to bridge the immunisation gaps and ensure that all children receive the recommended vaccines.^13^ This study aims to contribute to the existing literature by addressing the dearth of knowledge on absolute zero dose, antigen-wise zero dose, dynamics of the immunisation landscape and the immunisation cascade in India. This research aims to provide insights into these challenges and inform strategies that promote equitable access to vaccination services for all children in India, leaving no one behind.

## Methods

### Data

The NFHS-5 (2019–2021) provides information on population, well-being and nutrition with a demonstrative sample of individuals at a large scale. NFHS-5 is designed to be representative at the national, state and district levels. The survey aims to provide information on health and family welfare, like fertility levels, infant and child mortality, maternal and child health, and other health-related indicators by background characteristics. NFHS-5 also provides various kinds of health intervention indicators for children, such as vaccination of children, infant and young child feeding practices and utilisation of integrated child development services in India.

The NFHS-5 adopted a stratified two-stage sample design with the 707 districts as independent strata. First, the sample of primary sampling units (villages in rural areas and Census Enumeration Blocks (CEBs) in urban areas) was selected from the sampling frame with probability proportional to size. Second, a random systematic sampling technique was used to select the household of eligible women (15–49 years) from the village and CEBs in urban areas. NFHS-5 (2019–2021) collected information on health indicators from 724 115 women (urban—179 535, rural—544580) and 101839 men (urban—26 420, rural—75 419) from 636699 households (urban—160138, rural—476561) and 30198 PSUs.^31^

### Outcome and exposure variable

The outcome of interest is absolute zero dose and antigen-wise zero dose as defined

- Absolute zero dose refers to children who have not received a single dose of any routine vaccine by the age of 12–23 months. These children are entirely unvaccinated against preventable diseases targeted by routine immunisation programmes, making them particularly vulnerable to infections and contributing to gaps in population immunity.
- Antigen-wise zero dose refers to children who have not received any dose of specific vaccine within the routine immunisation schedule. Unlike absolute zero-dose children, who have not received any vaccines, antigen-wise zero-dose children may have received some dose of vaccines but completely missed uptake of certain individual antigens (eg, BCG, DPT, OPV and measles). This concept allows for a more granular analysis of immunisation gaps, helping to identify which specific vaccines have lower coverage among partially vaccinated children.

The exposure variables (socioeconomic and demographic characteristics) used were availability of the vaccination card (yes vs no), birth order (1, 2–3, 4 and more), children’s age (in months), wealth index (richest vs poorest vs poorer vs middle vs richer), sex of child (male vs female), social group, religion, residence (urban vs rural), mother education (in years), place of delivery (private facilities vs public facilities vs not in facilities), delivery by caesarean (yes vs no), media exposure of mother (yes vs no), residing with husband (yes vs no).

### Measure of immunisation landscape and immunisation cascade

The concept of the immunisation landscape talks about identifying key strengths (FIC), weaknesses (antigen-wise zero dose), opportunities (undervaccinated children) and threats (absolute zero dose) facing the country’s immunisation programme. The cascade characterises how, at the population level, infants move from zero dose to full vaccination coverage by describing which vaccines are most likely to be received by children who have had a single vaccine or combinations of two or more basic vaccines. This approach also provides more granular information on patterns of underimmunisation and drop-out across key vaccination touchpoints in the first year of life. This analysis focused on four vaccines, namely BCG, OPV, DPT-containing vaccine, and measles-containing vaccine (MCV).

### Statistical methods

**Figure 1:**
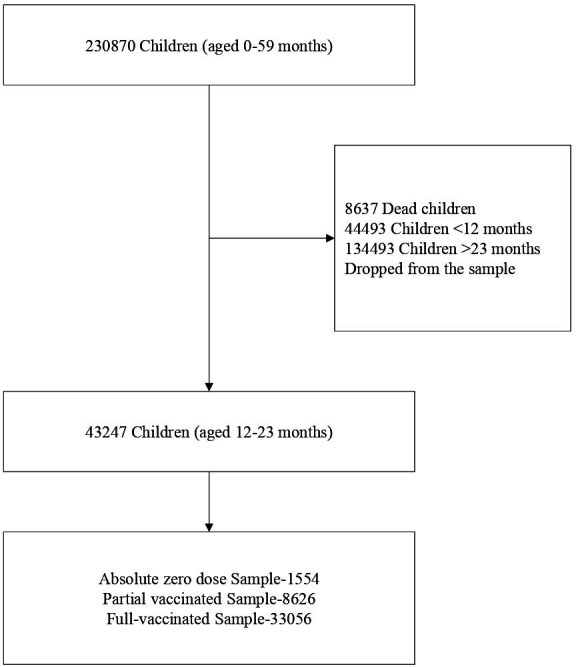
Analytical flow chart of the NFHS-5 Sample, 2019–2021. NFHS-5, National Family Health Survey.

The analytical flow of the analysis is shown in figure 1. Our outcome was a binary variable with a value of 1 if a child had absolute zero-dose vaccination and antigen-wise zero doses and 0 in all other cases. The absolute zero-dose vaccine is defined as the vaccine for children 12–23 months of age who have not received any routine vaccination. While partially vaccinated children mean children 12–23 months of age who have received at least one vaccine, but not taken all doses of the routine vaccines as per the National Immunisation Schedule. This study applied the bivariate analysis to determine the prevalence of absolute zero-dose and antigen-wise zero-dose children and to understand its distribution by explanatory variables. The bivariate analysis allows us to assess how the value of the outcome variable depends on the values displayed by the explanatory variable. We implemented the survey design effect to reduce the error estimation due to sampling stages and the sampling method, and analysis defaults, such as the method for variance estimation.

We used multilevel modelling to analyse data drawn from several levels and when our outcome is measured at the lowest level. We use the multivariate multilevel logistic regression to show the association at four levels (level 1: individual; level 2: PSU; level 3: district and level 4: state) with a 95% CI and p value. Before multilevel logistic regression, we checked multicollinearity between predictors; we used a generalised variance inflation factor, which usually should not exceed five; none of the predictors had a factor greater than five, which indicates there are no issues of multicollinearity. The hierarchical model of the survey justified the application of multilevel modelling in this study.


$$\begin{array}{l} logit\left(\pi_{icds})=log\left(\pi_{icds}\right)\right)/\;\left(1-\;\pi_{icds}\right)\\ \;=\beta_{0}+\;\beta_{1}x_{1icds}+\ldots \beta_{n}x_{nicds}\;+\alpha_{0cds}\;+v_{0ds}+u_{0s} \end{array}$$

Where $\pi_{icds}$ is the probability of children not receiving a binary outcome variable i in the cluster c, district d, and state level s ($\pi_{icds}$= 1 denotes success or the occurrence of the event, while $\pi_{icds}$= 0 denotes failure or lack of occurrence of the event). The parameter $\beta_{0}$is the intercept (mean) of the absolute zero dose among children 12–23 months old, and $\beta_{1}$represents the effects of the explanatory variables. The random intercepts regression model assumes that the intercept or average outcome for individuals with a given set of characteristics varies between higher-level units, and the relationship between the dependent and independent variables is consistent across all contexts. Randalpha, end base, sub c d s is the effect of the cluster, v sub d s is the effect of district, and $u_{s}$ is the effect of states level are the random effect or residual error term. The residual follows the assumption of independent and normal distribution with zero means and constant variances. The model estimates the variance at different levels: $\alpha_{cds}$ ∼ N (0,$\sigma_{c}^{2}$) is within the district, between cluster variance; $v_{ds}$ ∼ N (0, $\sigma_{d}^{2}$) is within states, between-district variance and $u_{s}$∼ N (0, $\sigma_{s}^{2}$) represents between-state variance.

The overall objective of multilevel models is to partition the variance in the outcome between the level of hierarchical data. The variance partition coefficient (VPC) is a statistical measure used to assess the proportion of variation in an outcome variable that can be attributed to different factors or sources of variation. It is commonly employed in the context of hierarchical or mixed-effects models, where there are multiple levels of nested data. VPC is the simple ratio of an area variance to the sum of the total level (1, 2 …N) of the variance in the outcome that is attributable to between-hierarchical structures variance. ^32^ The value of the variance of the underlying individual-level variable, according to the logistic distribution, is $\pi^{2}/3$ or 3.29.


$${VPC}_{g}=\frac{\sigma_{g}^{2}}{\left(\sigma_{s}^{2}+\sigma_{d}^{2}+\sigma_{c}^{2}+\pi^{2}/3\right)}$$

Where g represent a geographical area.

The Venn diagram approach shows the antigen-wise zero-dose vaccination among partially vaccinated children. In a Venn diagram, the intersection and union of overlapping concepts are presented. In India, among partially vaccinated children aged 12–23 months, a Venn diagram was used to examine the intersection between zero dosage of BCG, DPT, OPV and measles. Using a Venn diagram, you may visually depict sets and demonstrate the logical connections between them.

BCG Ո Measles+BCG Ո DPT+BCG Ո OPV+Measles ՈDPT+Measles Ո OPV+OPV Ո DPT+BCG Ո DPT Ո Measles+BCG Ո DPT Ո OPV+BCG Ո OPV Ո Measles+DPT Ո OPV Ո Measles. Where ∩ denotes intersection.

We also estimated the weighted prevalence of the absolute zero-dose and antigen-wise zero-dose vaccination among partially vaccinated children at the district level. NFHS-5 provides data on 707 districts of India. GIS was also used to show the prevalence of the absolute zero dose and antigen-wise zero-dose vaccination among partially vaccinated children for 707 districts of India.

## Result

Table 1 highlights the prevalence of absolute zero-dose children in India at 3.6%, with notable disparities across sociodemographic groups. Children without a vaccination card have the highest prevalence at 21.2%. Additionally, the prevalence is elevated among scheduled tribes (4.1%), Muslims (5.4%) and those in the poorest wealth quintile (4.6%). Maternal education plays a critical role; children whose mothers have no schooling show a 6.2% prevalence of zero-dose status, while this rate decreases to 2.7% among children of mothers with more years of schooling. Home deliveries and higher birth order also contribute to higher zero-dose prevalence, with 8.6% among children delivered at home and 6.1% for children of birth order four or higher. Lack of media exposure in mothers is associated with higher zero-dose prevalence (4.4%). Regarding partial immunisation, 20% of children in India are partially vaccinated, with the highest zero-dose rates for measles (42.8%), OPV (20.1%), pentavalent (13.7%) and BCG (5.9%), as shown in table 1. The analysis of sociodemographic factors reveals that children in the poorest quintile experience the highest zero-dose rates for BCG (8.2%), pentavalent (15.5%) and measles (46.9%), while OPV zero-dose prevalence peaks in the poorer quintile (22.3%). Scheduled tribe children also report elevated rates for BCG (8.6%), pentavalent (15.4%) and measles (48.1%), with Christians showing the highest rates of zero-dose for BCG, pentavalent, and OPV, and Muslims the highest for measles (51.6%).

**Table 1:** Prevalence of the absolute zero dose and antigen-specific zero dose among partially vaccinated children aged 12–23 months by socioeconomic variable in India, NFHS-5, 2019–2021

Background characteristic	Absolute zero dose of routine vaccination	BCG	DPT	OPV	Measles
**Vaccination card**	**% (95% CI**)	**% (95% CI**)	**% (95% CI**)	**% (95% CI**)	**% (95% CI**)
No	21.2 (19.5 to 23.1)	2.9 (2.3 to 3.6)	9.7 (8.5 to 11.1)	16.8 (15.2 to 18.5)	35.6 (33.2 to 38.1)
Yes	0.7 (0.6 to 0.8)	8.4 (7.3 to 9.6)	17.1 (15.6 to 18.6)	22.9 (21.3 to 24.5)	48.6 (46.6 to 50.5)
Birth order					
1	3.1 (2.7 to 3.4)	4.8 (3.9 to 5.9)	12.9 (11.4 to 14.7)	20.5 (18.6 to 22.5)	37.6 (35.2 to 40.0)
2–3	3.5 (3.1 to 4.0)	5.9 (5.0 to 6.9)	14.2 (12.7 to 15.7)	19.9 (18.3 to 21.5)	43.6 (41.4 to 45.9)
4 and more	6.1 (5.2 to 7.0)	8.8 (6.5 to 11.7)	14.4 (12.2 to 16.9)	20.2 (17.4 to 23.3)	52.7 (49.1 to 56.4)
Children age					
12–17 months	3.4 (3.1 to 3.7)	6.3 (5.4,7.4)	14.2 (12.9,15.6)	19.7 (18.2,21.3)	45.6 (43.5,47.7)
18–23 months	3.9 (3.4 to 4.4)	5.4 (4.6,6.5)	13.2 (11.8,14.8)	20.7 (19.0,22.4)	39.4 (37.3,41.6)
Wealth index					
Richest	3.2 (2.5 to 3.9)	8.2 (6.9 to 9.7)	15.5 (13.8 to 17.4)	21.1 (19.1 to 23.2)	46.9 (44.4 to 49.4)
Poorest	4.6 (4.1 to 5.1)	6.5 (5.0 to 8.3)	13.6 (11.8 to 15.7)	22.3 (20.0 to 24.7)	43.4 (40.7 to 46.3)
Poorer	3.9 (3.3 to 4.6)	4.7 (3.4 to 6.3)	12.4 (10.2 to 15.0)	17.6 (15.1 to 20.3)	41.4 (37.9 to 44.9)
Middle	3.2 (2.7 to 3.9)	3.6 (2.5 to 5.2)	12.7 (10.2 to 15.7)	18.4 (15.4 to 21.8)	42.2 (37.4 to 47.2)
Richer	2.8 (2.3 to 3.3)	4.6 (3.2 to 6.6)	13.1 (10.5 to 16.1)	20 (17.1 to 23.3)	35.7 (31.6 to 39.9)
Gender of child					
Male	3.3 (3.0 to 3.6)	5.9 (5.0 to 6.9)	13.5 (12.1 to 15.0)	20.5 (18.9 to 22.2)	42.5 (40.4 to 44.7)
Female	3.9 (3.5 to 4.5)	5.9 (5.0 to 7.0)	14 (12.7 to 15.5)	19.8 (18.2 to 21.4)	43 (40.9 to 45.1)
Social group					
None of them	3.8 (3.2 to 4.5)	6.5 (5.2 to 8.1)	13.6 (11.5 to 15.9)	19.5 (17.3 to 21.9)	40.6 (37.7 to 43.6)
Schedule caste	3.4 (2.8 to 4.1)	8.6 (6.5 to 11.2)	15.4 (12.6 to 18.6)	19.2 (16.2 to 22.6)	48.1 (42.3 to 54.0)
Schedule tribe	4.1 (3.5 to 4.8)	5.7 (4.7 to 6.9)	13.2 (11.8 to 14.7)	19.7 (18.1 to 21.4)	42.3 (40.2 to 44.4)
OBC	3.5 (3.2 to 3.9)	4.7 (3.5 to 6.2)	14.2 (12.1 to 16.7)	21.8 (19.3 to 24.6)	43.4 (40.2 to 46.8)
Religion					
Hindu	3.2 (3.0 to 3.5)	6.1 (5.4 to 6.9)	13.1 (12.0 to 14.3)	19.8 (18.5 to 21.1)	40.6 (38.9 to 42.4)
Muslim	5.4 (4.5 to 6.4)	4.9 (3.4 to 7.0)	16.6 (14.1 to 19.4)	21.8 (19.1 to 24.7)	51.6 (48.1 to 55.2)
Christian	4.5 (3.2 to 6.2)	7.4 (4.9 to 11.1)	18.2 (13.8 to 23.6)	22.7 (16.8 to 29.9)	46.1 (38.6 to 53.9)
Others	4.1 (2.1 to 7.9)	6.6 (3.5 to 12.0)	8.3 (5.5 to 12.4)	16.6 (12.1 to 22.4)	37.7 (30.4 to 45.7)
Residence					
Urban	4.4 (3.6 to 5.3)	4.5 (3.2 to 6.2)	15.5 (13.1 to 18.2)	19.3 (16.9 to 22.0)	42.3 (38.9 to 45.7)
Rural	3.3 (3.1 to 3.6)	6.4 (5.7 to 7.3)	13.1 (12.1 to 14.2)	20.4 (19.2 to 21.7)	42.9 (41.3 to 44.6)
Mother education in years					
>12 years	2.7 (2.2 to 3.3)	7.3 (5.8 to 9.0)	15.3 (13.5 to 17.4)	21.4 (19.2 to 23.8)	50 (47.2 to 52.8)
No schooling	6.2 (5.5 to 6.9)	10 (6.6 to 14.7)	13 (9.6 to 17.3)	17.7 (13.5 to 22.9)	45.5 (39.2 to 51.9)
<5 years	3.7 (2.9 to 4.9)	6.3 (5.1 to 7.8)	14.5 (12.6 to 16.6)	20.7 (18.6 to 23.0)	43.1 (40.4 to 45.9)
5–8 years	3.4 (2.7 to 4.3)	5 (3.9 to 6.2)	13.7 (11.9 to 15.8)	18.2 (16.3 to 20.3)	40.9 (38.1 to 43.7)
9–12 years	2.8 (2.4 to 3.2)	3.8 (2.6 to 5.4)	10.5 (8.3 to 13.1)	21.5 (18.2 to 25.2)	34 (29.3 to 39.1)
Place of delivery					
Private facilities	2.9 (2.6 to 3.2)	4.6 (3.9 to 5.3)	13.4 (12.2 to 14.8)	20.5 (19.1 to 22.0)	43.2 (41.3 to 45.0)
Public facilities	3.6 (3.1 to 4.2)	5.4 (4.2 to 7.0)	13.2 (11.2 to 15.5)	19.8 (17.6 to 22.2)	38.5 (35.1 to 42.0)
Not in facilities	8.6 (7.5 to 9.9)	13.2 (10.6 to 16.5)	16.3 (13.9 to 19.1)	19.2 (16.4 to 22.4)	50 (46.3 to 53.8)
Media exposure					
Yes	2.9 (2.5 to 3.2)	4.2 (3.4 to 5.1)	11.8 (10.4 to 13.5)	19.1 (17.3 to 21.0)	38.6 (36.2 to 41.2)
No	4.4 (4.0 to 4.8)	7.2 (6.3 to 8.4)	15.2 (13.9 to 16.7)	21 (19.5 to 22.5)	46 (44.1 to 47.9)
Delivery by caesarean					
No	3.9 (3.5 to 4.3)	6.3 (5.5 to 7.2)	14.2 (13.1 to 15.4)	20.3 (19.1 to 21.6)	44.2 (42.5 to 45.9)
Yes	2.7 (2.3 to 3.2)	4.3 (3.1 to 6.0)	11.9 (9.7 to 14.4)	19.4 (16.8 to 22.2)	37.1 (33.6 to 40.6)
Residing with husband					
Yes	3.6 (3.3 to 4.0)	5.7 (5.0,6.5)	13.4 (12.3,14.6)	19.9 (18.7,21.2)	42.7 (41.0,44.5)
No	3.7 (3.1 to 4.4)	6.8 (5.3,8.8)	15.4 (13.1,18.1)	21.1 (18.5,24.0)	42.8 (39.5,46.3)
Total	3.6 (3.3 to 3.9)	5.9 (5.2,6.6)	13.7 (12.8,14.8)	20.1 (19.0,21.3)	42.8 (41.2,44.3)
n/N	1564/43247	509/8626	1186/8626	1737/8626	3688/8626

DPT, diphtheria-tetanus-pertussis; NFHS-5, National Family Health Survey; OPV, oral polio vaccine.

The multivariate logistic regression model in table 2 further clarifies these findings. Children whose mothers possess a vaccination card are 0.03 times less likely to be absolute zero dose compared with those without (OR 0.03, 95% CI 0.02 to 0.03). Poverty increases the likelihood of zero-dose status, with children from the poorest households being 1.38 times more likely to be zero dose compared with the richest (OR 1.38, 95% CI 1.05 to 1.83). Social and religious groups also show disparities; scheduled tribe children are 1.26 times more likely to be zero dose than children from other social groups (OR 1.26, 95% CI 1.01 to 1.58), while Muslim and Christian children are 1.27 and 1.50 times more likely, respectively, to lack routine vaccination compared with Hindu children (OR 1.27, 95% CI 1.06 to 1.51; OR 1.5, 95% CI 1.10 to 2.06). Home deliveries are associated with a 1.62 times higher likelihood of zero-dose status than private facility births (OR 1.62, 95% CI 1.32 to 1.99).

**Table 2:** Multivariate multilevel logistic regression of absolute zero dose and antigen-wise zero dose among partially vaccinated children aged 12–23 months by socioeconomic variable in India, NFHS-5, 2019–2021

Background characteristic	Absolute zero dose of routine vaccination	BCG	DPT	OPV	Measles
Vaccination card	OR (95% CI)	OR (95% CI)	OR (95% CI)	OR (95% CI)	OR (95% CI)
No ^Ref.^	1	1	1	1	1
Yes	0.03*** (0.02, 0.03)	2.27*** (1.87, 2.76)	1.52*** (1.33, 1.74)	1.43*** (1.27, 1.62)	1.51*** (1.38, 1.66)
Birth order					
1 ^Ref.^	1	1	1	1	1
2–3	0.92 (0.81, 1.05)	1.18 (0.97, 1.44)	0.98 (0.85, 1.14)	0.9 (0.80, 1.02)	1.11* (1.00, 1.23)
4 and more	1.05 (0.88, 1.25)	1.24 (0.94, 1.62)	1.03 (0.84, 1.26)	0.91 (0.76, 1.09)	1.25** (1.08, 1.45)
Children age					
12–17 months ^Ref.^	1	1	1	1	1
18–23 months	1.05 (0.94, 1.18)	0.98 (0.83, 1.16)	0.95 (0.84, 1.08)	1.05 (0.94, 1.17)	0.84*** (0.77, 0.92)
Wealth index					
Richest ^Ref.^	1	1	1	1	1
Poorest	1.38* (1.05, 1.83)	1.31 (0.85, 2.03)	1.06 (0.78, 1.43)	1.02 (0.78, 1.33)	1.18 (0.94, 1.46)
Poorer	1.3 (1.00, 1.69)	1.21 (0.80, 1.83)	1.02 (0.77, 1.36)	1.15 (0.90, 1.47)	1.19 (0.97, 1.46)
Middle	1.17 (0.91, 1.51)	0.99 (0.66, 1.50)	0.95 (0.72, 1.26)	0.93 (0.73, 1.19)	1.12 (0.92, 1.36)
Richer	1.15 (0.90, 1.47)	0.93 (0.62, 1.39)	0.92 (0.70, 1.20)	0.93 (0.74, 1.17)	1.18 (0.97, 1.42)
Gender of child					
Male ^Ref.^	1	1	1	1	1
Female	1.04 (0.93, 1.16)	1.09 (0.92, 1.28)	1.06 (0.93, 1.20)	0.96 (0.86, 1.08)	1.02 (0.93, 1.11)
Social group					
None of them ^Ref.^	1	1	1	1	1
Schedule caste	0.98 (0.81, 1.19)	1.25 (0.93, 1.70)	0.92 (0.74, 1.15)	0.85 (0.70, 1.02)	0.98 (0.84, 1.14)
Schedule tribe	1.26* (1.01, 1.58)	1.25 (0.89, 1.76)	1.02 (0.80, 1.31)	1.02 (0.82, 1.27)	1.04 (0.87, 1.25)
OBC	1.03 (0.87, 1.22)	1.1 (0.84, 1.44)	0.86 (0.72, 1.03)	0.84* (0.72, 0.99)	0.97 (0.85, 1.11)
Religion					
Hindu ^Ref.^	1	1	1	1	1
Muslim	1.27** (1.06, 1.51)	0.75* (0.56, 0.99)	1.19 (0.99, 1.44)	1.09 (0.92, 1.30)	1.48*** (1.29, 1.70)
Christian	1.50* (1.10, 2.06)	1.03 (0.67, 1.59)	1.39* (1.04, 1.84)	1.02 (0.75, 1.40)	1.39* (1.07, 1.80)
Others	1.11 (0.79, 1.58)	1.18 (0.73, 1.90)	0.91 (0.63, 1.31)	1.11 (0.80, 1.56)	0.99 (0.75, 1.30)
Residence					
Urban ^Ref.^	1	1	1	1	1
Rural	0.80** (0.68, 0.94)	0.97 (0.75, 1.26)	0.75** (0.62, 0.90)	0.96 (0.82, 1.13)	0.84** (0.73, 0.95)
Mother education in years					
>12 years ^Ref.^	1	1	1	1	1
No schooling	1.33* (1.04, 1.70)	0.98 (0.66, 1.44)	1.2 (0.91, 1.58)	0.97 (0.76, 1.23)	1.50*** (1.23, 1.82)
<5 years	1.15 (0.85, 1.56)	1.18 (0.74, 1.88)	1.09 (0.76, 1.56)	0.99 (0.73, 1.35)	1.30* (1.01, 1.68)
5–8 years	1.02 (0.81, 1.29)	1.03 (0.72, 1.49)	1.17 (0.91, 1.52)	0.94 (0.76, 1.16)	1.30** (1.09, 1.56)
9–12 years	1.01 (0.82, 1.24)	1.05 (0.74, 1.47)	1.06 (0.83, 1.35)	0.86 (0.71, 1.05)	1.23* (1.05, 1.46)
Place of delivery					
Private facilities ^Ref.^	1	1	1	1	1
Public facilities	0.87 (0.74, 1.03)	0.77 (0.59, 1.00)	0.94 (0.78, 1.14)	0.92 (0.78, 1.07)	1.11 (0.98, 1.27)
Not in facilities	1.62*** (1.32, 1.99)	2.15*** (1.58, 2.93)	1.17 (0.92, 1.49)	0.70** (0.56, 0.88)	1.38*** (1.15, 1.64)
Media exposure					
Yes ^Ref.^	1	1	1	1	1
No	1.12 (0.98, 1.28)	1.22 (0.99, 1.50)	1.18* (1.01, 1.38)	1.04 (0.91, 1.19)	1.15* (1.03, 1.28)
Delivery by caesarean					
No ^Ref.^	1	1	1	1	1
Yes	1.01 (0.85, 1.20)	1.08 (0.82, 1.43)	0.88 (0.72, 1.07)	0.91 (0.77, 1.08)	0.93 (0.81, 1.06)
Residing with husband					
Yes ^Ref.^	1	1	1	1	1
No	0.95 (0.81, 1.12)	1.18 (0.93, 1.50)	1.1 (0.91, 1.32)	1.03 (0.87, 1.21)	1.03 (0.90, 1.17)

* p<0.05, **p<0.01, ***p<0.001.

DPT, diphtheria-tetanus-pertussis; NFHS-5, National Family Health Survey; OPV, oral polio vaccine.

The detailed regression model in table 2 also assesses antigen-specific zero-dose factors among partially vaccinated children. Children with vaccination cards have significantly higher odds of zero-dose status for BCG, DPT, OPV and Measles. Additionally, Muslim children are 0.75 times less likely to report zero-dose BCG yet more likely to miss DPT and Measles vaccinations compared with Hindu children. Rural children are less likely to be zero-dose for DPT and Measles than their urban counterparts. Maternal education impacts measles zero-dose prevalence substantially; children of mothers without schooling show a 50% prevalence, while those with mothers with 12+ years of education show only a 34% prevalence, a significant 16% reduction.

In table 3, variance partitioning indicates that 3.69% of absolute zero-dose variation occurs between states, 5.31% within states between districts and 5.67% within districts between clusters. For partially vaccinated children, antigen-wise zero-dose variance reveals that 4.09%, 3.21% and 2.50% of BCG, DPT and measles zero-dose variation can be attributed to state-level differences. District-level differences contribute 2.24% and 2.82% to OPV and measles zero-dose prevalence, while cluster-level variation within districts significantly affects DPT and OPV zero-dose rates.

**Table 3:** Multilevel distribution of the variance partition coefficients (VPCs) of the absolute zero-dose vaccine and antigen-wise zero-dose vaccine among partially vaccinated children aged 12–23 months in India, NFHS-2021

Geographies	Absolute zero dose	Antigen-wise zero dose among partially vaccinated children _			
		BCG	DPT	OPV	Measles
	VPC, (95% CI)	VPC, (95% CI)	VPC, (95% CI)	VPC, (95% CI)	VPC, (95% CI)
States	3.69, (1.26 to 5.72)	4.09, (0.71 to 7.01)	0.27, (−0.39 to 0.82)	3.21, (1.08 to 5.01)	2.50, (0.82 to 3.99)
District	5.31, (3.50 to 6.80)	4.98, (1.36 to 8.11)	2.87, (0.77 to 4.64)	2.24, (0.63 to 3.60)	2.82, (1.57 to 3.92)
Cluster	5.67, (1.26 to 9.33)	0.00, (0.00 to 0.00)	10.58, (5.18 to 15.12)	10.67, (6.74 to 14)	2.24, (−0.93 to 5.04)

NFHS, National Family Health Survey; OPV, oral polio vaccine.

**Figure 2:**
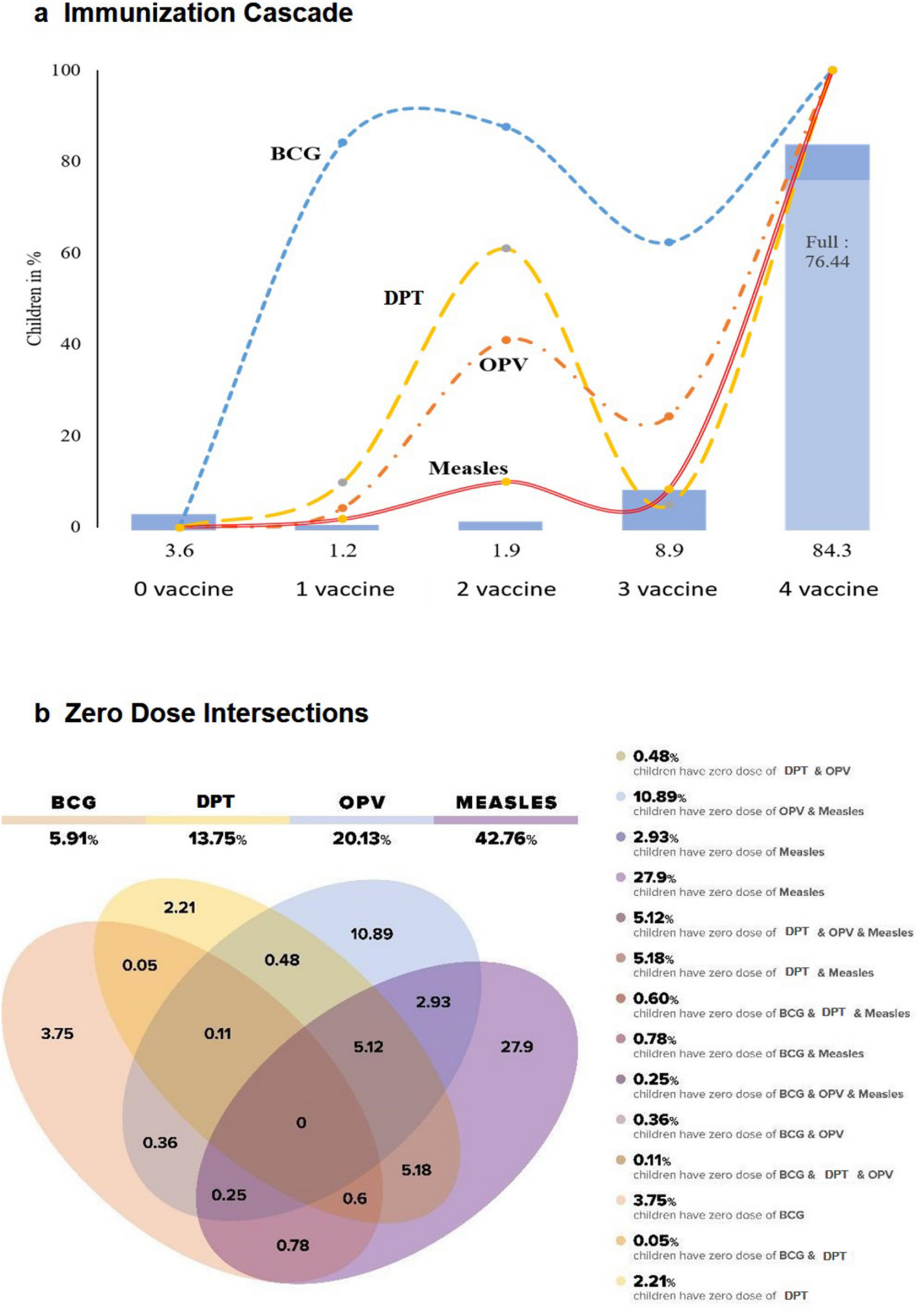
Immunisation cascade and antigen-wise zero-dose intersections among partially vaccinated children aged 12–23 Months in India, NFHS-5 (2019–2021). (a) Immunisation cascade, (b) zero-dose intersections. DPT, diphtheria-tetanus-pertussis; NFHS, National Family Health Survey; OPV, oral polio vaccine.

Figure 2b depicts the visual overlapping of antigen-wise zero dose among partially vaccinated children. There were four ellipses of zero-dose vaccine as: BCG, DPT, OPV and measles. Among the partially vaccinated children, 42.76% of children are zero doses for measles, 20.13% for OPV, 13.75% for DPT and 5.91% for BCG. The children with antigen-wise zero dose, 3.75% BCG, 2.21% DPT, 10.89% OPV and 27.90% measles did not overlap with each other among partially vaccinated children 12–23 months old in India. The two or three shaded portions of the ellipses show the intersection of sets of antigen-wise zero-dose children.

The table 4 and figure 2a show the co-coverage and cascade level of all possible combinations of vaccines in each cascade level. In India, 84.29% of children aged 12–23 months received at least one dose of all vaccines (BCG+one dose of DPT+one dose of OPV+one dose of measles vaccine). The figure also shows that 76.4% of children received all doses of the four vaccines, that is, fully immunised. The ratio difference between the two estimates suggests that 9.3% of children who receive BCG, MCV and at least the first dose of OPV and DPT vaccines do not progress to being fully vaccinated against polio and DPT. Among children who received at least two different vaccines, BCG and OPV (1.03%) were the most frequent combination, and for children who received three different vaccines, BCG, OPV and DPT (5.56%) were the most common combination.

**Table 4:** Co-coverage with four vaccines and cascade levels of undervaccination due to missed doses among children aged 12–23 months in India: NFHS-5 (2019–2021)

Cascade level		Vaccines combinations		% Coverage	N
0 vaccines		0 vaccination		3.62	1564
1 vaccine		BCG		1.02	441
		DPT		0.05	22
		OPV		0.12	52
		MCV		0.02	10
2 vaccines		BCG+DPT		0.58	253
		BCG+OPV		1.03	447
		BCG+MCV		0.09	41
		DPT+OPV		0.16	67
		DPT+MCV		0.07	32
		OPV+MCV		0.01	5
3 vaccines		BCG+DPT+OPV		5.56	2407
		BCG+DPT+MCV		2.17	938
		BCG+OPV+MCV		0.44	191
		DPT+OPV+MCV		0.75	324
4 vaccines		BCG+DPT+OPV+MCV		84.29	36 454
**Missed any one dose**		**Missed any two dose**		**Missed any three dose**	
BCG	0.64	OPV2+OPV3	1.10	OPV1+OPV2+OPV3	1.73
DPT1	0.17	DPT2+DPT3	0.15	DPT3+OPV3+MCV1	1.23
DPT2	0.09	DPT3+OPV3	0.68	DPT3+OPV2+OPV3	0.23
DPT3	0.51	DPT3+MCV1	0.39	OPV1+OPV2+MCV1	0.38
OPV1	0.29	OPV3+MCV1	0.79		
OPV2	0.20	OPV1+OPV2	0.05		
OPV3	3.91	DPT2+OPV2	0.03		
MCV1	0.85	OPV1+OPV3	0.06		
		DPT1+DPT3	0.04		
Total	6.70%	Total	3.29%	Total	3.84%

DPT, diphtheria-tetanus-pertussis; MCV, measles-containing vaccine; NFHS-5, National Family Health Survey; OPV, oral polio vaccine.

The findings of the table 4 also show the percentage of children who missed achieving the status of FIC due to the number of missed doses. The table shows the percentage of children who missed one, two and three doses of the vaccine. Overall, in India, out of 20% of the partially vaccinated children, 7.34% of children missed one, two or all three dose of the OPV vaccine. In India, 6.7% of children could not complete the full course of vaccination by their first year of life as they missed one dose of the vaccine. Most of such children missed the OPV3 dose 3.9%. The second shows that 3.29% of children missed the FIC status due to missing any two doses of the vaccine, and in this category also, most of the children missed the OPV second dose and OPV third dose 1.10%. In the third category, 3.84% of children missed any three doses of the vaccine, and in this category, also most of the children, 1.73% missed all three doses of OPV.

**Figure 3:**
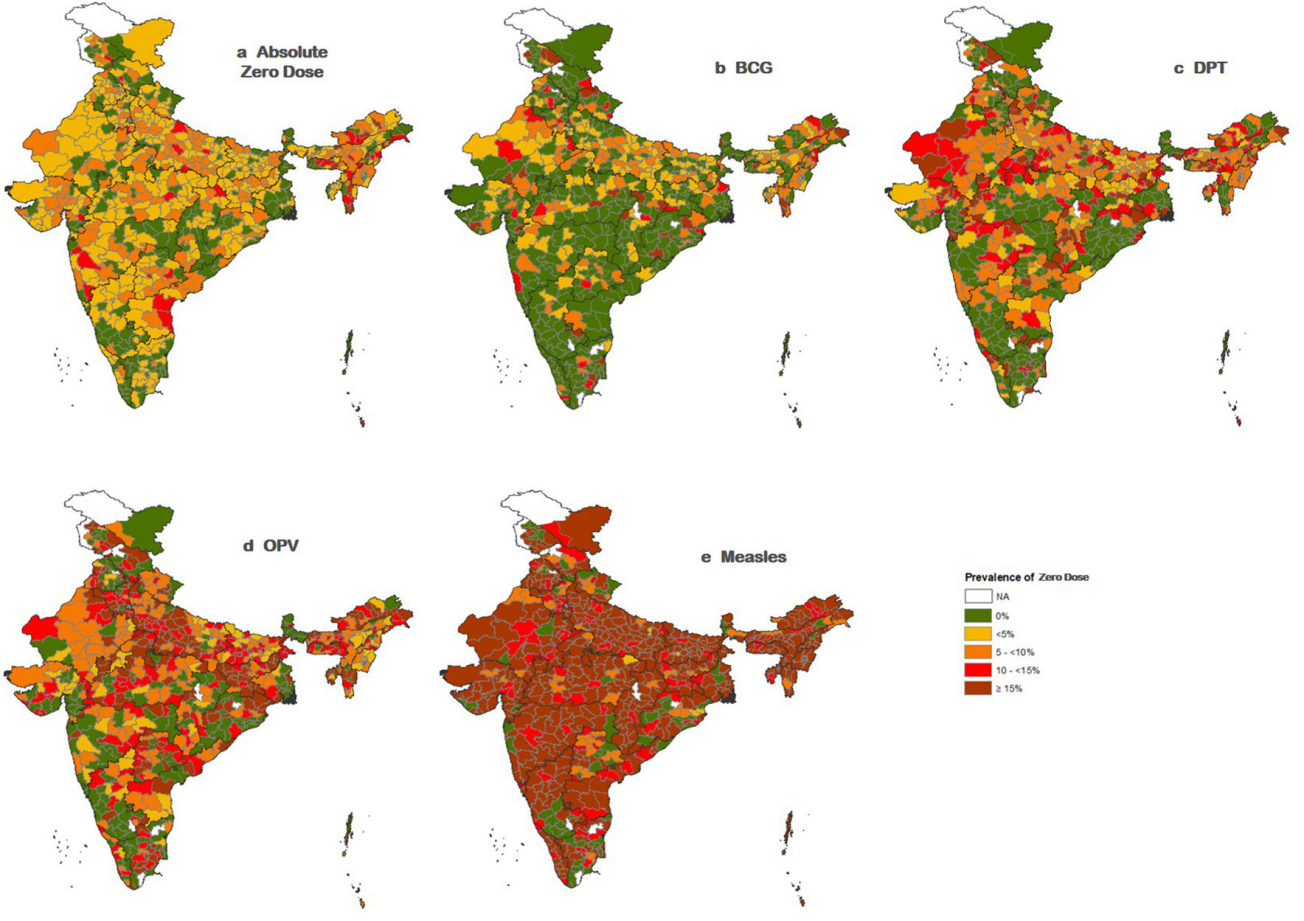
Prevalence of absolute and antigen-specific zero doses of routine vaccination among children aged 12–23 months in India, 2019–2021. DPT, diphtheria-tetanus-pertussis; OPV, oral polio vaccine.

Figure 3a shows the prevalence of absolute zero dose (left-out) of routine vaccination among children aged 12–23 months in 707 districts of India. In addition, figure 3b-e reveals the antigen-wise zero dose of the vaccination. All maps in figure 3 show a high prevalence of absolute zero dose and antigen-specific zero dose in the central and northeastern part of India.

## Discussion

The findings of this study provide significant insights into the immunisation landscape in India, specifically regarding the completion of the immunisation schedule. These findings underscore the need to address the issue of being left out (absolute zero dose) and incomplete vaccination (antigen-wise zero dose) and highlight the importance of reaching out to these undervaccinated (partially immunised) children to ensure they receive the full range of recommended vaccines as per the National Immunisation Schedule. The findings highlight that the prevalence of left out (absolute zero dose) and antigen-wise zero-dose children are higher among mothers having no schooling, Muslim religion, children belonging to an urban region, children delivered at home and children from the poorest wealth quintile. These findings align with some of the work related to immunisation. 12 10,12 33 The antigen-wise zero dose among partially vaccinated subset children OPV zero dose shows a different pattern. The antigen-wise zero dose highlights the highest prevalence of measles zero dose, followed by OPV zero dose. The cascade finding also highlights that 84.29% of children in India have received at least one dose of all vaccines. One of the key findings of this study is the substantial percentage of children in India who are unable to complete the scheduled doses of vaccines, resulting in partial immunisation. These findings emphasise the need to address the issue of incomplete vaccination and the importance of reaching out to these undervaccinated children to ensure they receive the full range of recommended vaccines. A particular concern arises regarding the coverage related to the OPV. In total, 7.34% of children fall into the partially vaccinated category due to being under or unvaccinated (OPV zero dose) for OPV. The findings align with other work on OPV, which has a high drop-out rate compared with other antigens. Strengthening OPV coverage and addressing the data associated challenges are vital to improving immunisation coverage rates.^33^

By using a multilevel random effect analysis, the findings also shed light on the influence of geographical areas on the variation in absolute zero dose and antigen-wise zero dose among children aged 12–23 months in India. The results indicate that spatial variation is notably high for absolute zero dose and zero dose of DPT and OPV at the cluster level. In contrast, variation is more pronounced between districts for BCG and Measles. These findings underscore the importance of enhancing routine vaccination coverage through health administrative measures at the micro level rather than macro approaches, particularly at the cluster and district levels. By doing so, we can effectively reduce the likelihood of absolute zero dose and undervaccination among children aged 12–23 months in India. Furthermore, the study’s unique contribution lies in its ability to meticulously map the vaccination history of partially immunised children, elucidating both the vaccines they have received and those that have been missed. This mapping provides valuable insights for targeted intervention strategies and reinforces the urgency of addressing the gaps in immunisation coverage.

Along with zero doses, addressing barriers to underimmunisation is essential for improving equity in coverage, particularly benefiting disadvantaged children who are more likely to be zero-dose or experience higher drop-out rates.^1 4 34 35^

This can be achieved through various strategies, such as improved communication and education about the importance of completing the immunisation schedule, strengthening healthcare delivery systems and addressing any barriers that may prevent children from accessing the required doses.

The study also highlights the proportion of children in India who cannot complete the immunisation schedule due to missing one, two, or three doses of vaccines. For instance, 6.7% of children cannot complete the immunisation schedule due to missing any one dose of vaccine, while 3.29% have missed any two doses, and 3.84% have missed three doses. It is important to note that an individual is considered fully immunised if they receive a total of eight doses across four vaccines. These missed children represent a group that can be easily targeted for interventions to ensure they receive the remaining doses and achieve full immunisation. Addressing incomplete immunisation among these groups presents a significant opportunity to improve immunisation coverage rates in India.

Furthermore, the Venn diagram highlights the importance of focusing and reducing the measles zero-dose children because measles is the last vaccine given to children to be classified as fully vaccinated before 12 months. Measles zero dose can be considered an early warning sign for immunisation programmes. They can be effectively used as a signal for tracing missed and drop-out children and strengthening the overall system for a universal immunisation programme. The reduction in measles zero doses will ensure the child has received all the past scheduled doses of DPT, BCG and OPV.^13 28 37^ By converting these low-hanging fruit into fully immunised individuals, significant progress can be made towards achieving universal immunisation coverage beyond 95% in India. It is noteworthy that a recent policy update has played a solid role in catching up with the absolute zero dose and drop-out children. The efforts made through programmes like Mission Indradhanush and Intensified Misson Indradhanush (IMI), a periodic catchup campaign launched in 2014 (IMI) and e-VIN implementation have contributed to bridging the immunisation gaps,maintaining timely vaccine stocks, strengthening service delivery and reaching children who were previously missed.^40 41^According to the NFHS data, the FIC rate increased from 62% in NFHS-4 (2015–2016) to 76.4% in NFHS-5 (2019–2021). Furthermore, partially immunised children decreased from 30% in NFHS-4 to 20% in NFHS-5, while the proportion of absolute zero-dose children decreased from 6.2% to 3.6%.^29 30^ These statistics indicate that the policies and plans implemented in India are moving in the right direction to reduce the number of absolute zero-dose children and decrease the percentage of partially vaccinated children. These improvements in immunisation coverage rates reflect the country’s commitment to the IA 2030 and the goal of leaving no child behind. One missing link might be to cater the immunisation coverage among home delivery children through different touch points along with extensive and targeted counselling of mothers regarding institutional delivery, immunisation and its benefits in the short and long term. Furthermore, the husband and in-laws’ support and involvement in maternity services, children’s health in general and timely immunisation services in particular is crucial to achieving the IA 2030.

### Limitations

This study also has a few limitations. The first notable part of the data on child immunisation was reported via caregiver recall, which may result in misclassifying the immunisation landscape and immunisation cascade due to the recall bias. The second is the low coverage of OPV as compared with injectable penta-containing vaccines, which are given together in the same immunisation schedule at the same session site. The reasons need to be explored. The low coverage of OPV may also result from data recording and under-reporting of the OPV vaccine, and the current study is unable to explore the potential reasons for low OPV coverage due to data limitations. Further qualitative research is needed to explain the reasons for absolute zero dose, low OPV and measles vaccine coverage, and antigen-wise zero dose among partially immunised children.

## Conclusions

The study findings underscore the need to address incomplete vaccination in India, with a considerable percentage of children missing one, two, or three doses of vaccines. Strengthening OPV and measles vaccine coverage and addressing challenges related to data recording and reporting practices are crucial steps towards improving immunisation coverage rates. Efforts focused on specific populations to ensure they receive all vaccine doses and achieve full immunisation are required. The study findings also shed light on the patterns of partially vaccinated children by highlighting antigen-wise zero dose in India. Understanding which vaccines are missed among partially vaccinated children enables the development of targeted interventions to close these gaps effectively. Moreover, sociodemographic factors, which have emerged as major determinants of vaccine uptake, need to be addressed to improve FIC. The immunisation programme can plan to use these associated factors as a point of entry to identify potential gaps and try to design specific strategies to reach these groups.

Thus, targeted interventions must take a holistic approach that covers all women and children through continuous counselling of mothers, husbands and in-laws through Accredited Social Health Activist (ASHA) and Auxiliary Nurse Midwife (ANM), which should be an integral part of the universal immunisation programme. This also re-emphasises the need to have an integrated approach, that is, the availability of immunisation services with primary healthcare services, which have more accessibility in periurban, rural and remote areas and achieve the IA 2030.

## Data Availability

Data are available in a public, open access repository.
